# Caloric restriction improves glycaemic control without reducing plasma branched-chain amino acids or keto-acids in obese men

**DOI:** 10.1038/s41598-022-21814-z

**Published:** 2022-11-11

**Authors:** M. H. Sayda, M. H. Abdul Aziz, N. Gharahdaghi, D. J. Wilkinson, P. L. Greenhaff, B. E. Phillips, K. Smith, I. Idris, P. J. Atherton

**Affiliations:** 1 MRC-Versus Arthritis Centre for Musculoskeletal Ageing Research, University of Nottingham, Derby, UK; 2 Nottingham NIHR Biomedical Research Centre, Nottingham, UK; 3 National Centre for Sport and Exercise Medicine, Nottingham, UK; 4 Division of Medical Sciences and Graduate Entry Medicine, University of Nottingham, Royal Derby Hospital Centre, Derby, DE22 3DT UK

**Keywords:** Biochemistry, Diseases, Endocrinology, Medical research

## Abstract

Higher plasma leucine, isoleucine and valine (BCAA) concentrations are associated with diabetes, obesity and insulin resistance (IR). Here, we evaluated the effects of 6-weeks very-low calorie diet (VLCD) upon fasting BCAA in overweight (OW) non-diabetic men, to explore associations between circulating BCAA and IR, before and after a weight loss intervention. Fasting plasma BCAAs were quantified in an OW (*n* = 26; BMI 32.4 ± 3 kg/m^2^; mean age 44 ± 9 y) and a normal-weight (NW) group (*n* = 26; BMI 24 ± 3.1 kg/m^2^; mean age 32 ± 12.3 y). Ten of the OW group (BMI 32.2 ± 4 kg/m^2^; 46 ± 8 y) then underwent 6-weeks of VLCD (600–800 kcal/day). Fasting plasma BCAA (gas chromatography-mass spectrometry), insulin sensitivity (HOMA-IR) and body-composition (DXA) were assessed before and after VLCD. Total BCAA were higher in OW individuals (sum leucine/isoleucine/valine: 457 ± 85 µM) compared to NW control individuals (365 ± 78 µM, *p* < 0.001). Despite significant weight loss (baseline 103.9 ± 12.3 to 93 ± 9.6 kg and BMI 32.2 ± 4 to 28.9 ± 3.6 kg/m^2^), no changes were observed in BCAAs after 6-weeks of VLCD. Moreover, although VLCD resulted in a significant reduction in HOMA-IR (baseline 1.19 ± 0.62 to 0.51 ± 0.21 post-VLCD; *p* < 0.001), Pearson’s r revealed no relationships between BCAA and HOMA-IR, either before (leucine R^2^: 2.49e−005, *p* = 0.98; isoleucine R^2^: 1.211−e006, *p* = 0.9; valine R^2^: 0.004, *p* = 0.85) or after VLCD (leucine R^2^: 0.003, *p* = 0.86; isoleucine R^2^: 0.006, *p* = 0.82; valine R^2^: 0.002, *p* = 0.65). Plasma BCAA are higher in OW compared to NW individuals. However, while 6-weeks VLCD reduced body weight and IR in OW individuals, this was not associated with reductions in BCAA. This suggests that studies demonstrating links between BCAA and insulin resistance in OW individuals, are complex and are not normalised by simply losing weight.

## Introduction

The global prevalence of obesity (BMI of ≥ 30 kg/m^2^) has reached epidemic proportions and is predicted to rise from 33% in 2005 to ~ 58% by 2030^1^. Obesity is a central feature of insulin resistance (IR), a major risk factor for developing type 2 diabetes (T2DM;^2,3^), whilst also being associated with adverse outcomes to multiple co-morbidities, including COVID-19^3^. Thus, novel insights into the pathophysiology of IR, and effective treatments are needed.

Branched-chain amino acids (BCAA) are diet-derived nutrients, which are established regulators of skeletal muscle proteostasis that act as critical anabolic signals [and substrates] positively regulating muscle and whole-body protein synthesis^4,5^. Yet, excess circulating BCAA, and their keto-acid by-products^6,7^, have been associated with adverse metabolic health; for example several studies have shown that dietary BCAA restriction^8^ and pharmacological reduction of plasma BCAAs^9^ can improve insulin sensitivity (IS). Over decades, studies have consistently shown that higher blood concentrations of BCAAs are common diagnostic and prognostic features for IR and risks of T2DM, with historical^10^, and more recent data^11,12^, proposing BCAA to be a root-cause of IR and dysglycaemia across liver, skeletal muscle and adipose tissue sites indepedent of BMI^13,14^. The proposed mechanisms by which BCAAs induce insulin resistance centre upon two themes: (1) that excess dietary BCAAs lead to sustained activation of mTORC1 (of which, leucine is a trigger^15^) via serine phosphorylation of insulin receptor substrate (IRS-1) and IRS-2, and that (2) impaired BCAA metabolism results in accumulation of BCAA-metabolic intermediates which suppress insulin action resulting in lipid accumulation^11^. Some studies have also shown reduced skeletal muscle expression of genes involved in BCAA metabolism in those with T2DM^16^, whilst others have shown that insulin-resistant rats demonstrate reduced capacity for BCAA catabolism^17^. Finally, work from our lab^18^ has shown that treating murine C2C12 myoblasts with sodium phenylbutyrate (PB), which induces BCAA catabolism, reduces BCAA and branched-chain keto-acid (BCKA) concentrations, impaires IRS-1 signaling with concomitant increases in phosphorylation of protein kinase B (AKT). In sum, multiple lines of evidence point to dysregulated BCAA metabolism in insulin resistant states.

However, a general limitation of this research area is a lack of “cause-effect” relationships between higher BCAA and IR in overweight humans (OW)—an aspect which could be addressed in longitudinal weight loss studies tracking BCAA abundance. Of all means to induce weight loss, non-pharmaceutical approaches remain an important first step in the management of obesity and diabetes. Specifically, very low-calorie diets (VLCD) represent a safe and effective means to reduce IR with short-term (~ 8 week) VLCD being sufficient to normalise hepatic IR, improve β-cell function in individuals with T2DM^19^ and induce remission of T2DM^20^, whilst also proving efficacious in obese^21^ and adolescent populations^22^. The aim of this study was to investigate relationships between fasting plasma BCAA/BCKA and insulin sensitivity (IS) following 6-weeks of VLCD in OW men. We hypothesised that BCAA would be higher in our obese volunteers, and that ensuing weight loss and reductions in IR would be associated with a normalisation of BCAA, that would further be associated with improved IS.

## Research design and methods

### Ethical approval

This study was approved by the University of Nottingham Faculty of Medicine Ethics Committee (D/2/2006-B12092016) and complied with the 2013 Declaration of Helsinki. Informed consent was obtained from all participants prior to enrolment onto the study.

### Participant characteristics

To confirm that OW individuals have increased fasting plasma BCAA concentrations, we undertook baseline measurements of OW men (*n* = 26, 44 ± 9 y, BMI 32.4 ± 3 kg/m^2^) and NW individuals (*n* = 26 {8 female: 18 male} , 32 ± 12.3 y, BMI 24 ± 3.1 kg/m^2^). The samples from the latter cohort were obtained from participants in a previously published study from our laboratory^23^. Of the twenty-six OW individuals, *n* = 10, middle-aged men (45.9 ± 8.3 y, BMI 32.2 ± 4 kg/m^2^) of mixed ethnic backgrounds underwent 6-weeks VLCD. Volunteers with diabetes, hypertension, respiratory or cardiovascular disorders were excluded. Individuals were not taking any prescribed medications. Before enrolment, participants were screened by a medical questionnaire and physical examination (resting 12-lead ECG, clinical blood chemistry).

### Study procedures

On study days, volunteers reported to our laboratory at ~ 09:00 h fasted (except water) from midnight. Body composition was determined by dual-energy X-ray absorptiometry (DXA; Lunar Prodigy II, GE Medical Systems) with body regions auto-processed (Encore software, GE Healthcare). Fasting blood samples were collected from the antecubital vein into lithium-heparin (for plasma) or fluoride oxalate (for glucose) vacutainers, before centrifugation at 3500 rpm, at 4 °C for 20 minutes, wherein the plasma was then rapidly frozen and stored at − 80 °C.

### Dietary management

VLCD study participants (*n* = 10), were instructed to maintain their usual levels of physical activity and prescribed a meal replacement diet designed to aid in weight management (Lighter Life, Harlow, Essex, U.K). This consisted of 4 meals per day, providing ~ 600 kcal/day, with an allowance for an extra 200 kcal/day in the form of fruit, vegetables or meat. The meals provided approximately 50 g protein, 50 g carbohydrate and 17.3 g fat, complete with 100% RDA of vitamins and minerals. 

#### Analytical methods

To determine plasma BCAA and BCKA concentrations, samples were spiked with internal standards (Norleucine and α-Ketovaleric acid) and prepared according to our routine methods^15^. Briefly, plasma proteins were precipitated in 100% ice-cold ethanol, then pelleted by centrifugation at 10,000 rpm for 3 min at 4 °C; the supernatant was dried under N_2_ at 90 °C in a Techne Dri-Block. The Quinoxalinol derivative of the BCKA and KVA (internal standard) were prepared using acidified 0.15% *ortho*-phenylenediamine (OPD) in 1.3 M HCl, at 90 °C for 60 min, then allowed to cool. Lipids and the BCKA-quinoxalinol derivatives were removed by extraction into ethyl acetate. The ethyl acetate layer and the remaining aqueous layer (containing the BCAA), were dried down separately and the N-tert-butyldimethyl-silyl-N-methyl-trifluoracetamide (MTBSTFA) derivatives of each fraction (BCKA and KCAA) were prepared, and their concentrations were then determined separately by GC–MS. This involved using selected ion-monitoring (SIM) approaches, monitoring m/z 288 (Val), 302 (Leu, Ile and Norleu), and m/z 245 (KIV and KVA) and 259 (KIC and KMV). Concentrations of plasma BCAA and BCKA were determined with reference to a standard curve of known concentrations. A pooled plasma quality control (QC) sample (study-specific sample) was prepared and analysed throughout each batch to monitor instrument performance.

#### GC–MS conditions

Approximately 0.5 µl of each fraction was injected into an ISQ Trace 1300 single quadrupole GC–MS (ThermoFisher Scientific, Hemel Hempstead, UK). Split injection mode (1:10) was used, with an initial oven temperature of 100 °C, held for 1 min, then ramped at 12 °C/min to 300 °C, held for 5 min to ensure elution of higher boiling compounds. Helium was the carrier gas at a flow rate of 1.5 mL/ min, and separation was achieved on a 30 m Rxi-5MS (0.25 mm internal diameter, 0.25 µm thickness) fused-silica column (Restek, Bellafonte, Pennsylvania).

#### Insulin and glucose concentrations

Plasma insulin and glucose, were assessed in duplicate^23^. Insulin was assessed via a high-sensitivity human insulin ELISA (DRG Instruments GmbH) according to manufacturer’s instructions. Glucose was measured using a clinical chemistry analyser (YSI 2950 Biochemistry analyser, YSI LifeSciences, Ohio, USA) against commercial standards. IS was calculated via HOMA-IR, according to the formula:

$$ \begin{aligned} & \left( {fasting\;plasma\;glucose\;concentration\;({\text{mmol}}.{\text{l}}^{ - 1} )} \right. \\ & \quad \left. { \times fasting\;plasma\;insulin\;concentration\;({\text{mU}}.{\text{l}}^{ - 1} )} \right)/22.5 \\ \end{aligned} $$

### Statistical analysis

Based on normality of data or transformation where required, BCAA and BCKA concentrations were compared between OW and NW controls using Student’s unpaired *t*-tests. Changes in BCAA concentrations in OW participants who underwent 6-weeks of VLCD were compared using Student’s paired *t*-test. The relationship between HOMA-IR and BCAA at baseline and following VLCD was investigated using Pearson’s correlation. All data are presented as mean ± SEM with significance at *p* < 0.05. Analyses were performed using GraphPad Prism v8.3 (La Jolla, CA, USA).

## Results

### Effects of VLCD on body composition and circulating BCAA levels

Six weeks VLCD elicited reductions in whole-body mass (baseline 103.9 ± 12.3 kg, post-VLCD 93 ± 9.6 kg; *p* < 0.001) and BMI (baseline 32.2 ± 4, post-VLCD 28.9 ± 3.6 kg/m^2^; *p* < 0.001), with concomitant declines in whole-body lean mass (baseline 65.2 ± 6 kg, post-VLCD 60.9 ± 4.8; *p* < 0.001), Table 1.

**Table 1:** Changes of parameters in participants who underwent VLCD (*n* = 10), variables derived from DXA and statistical significance achieved via paired *t-*test.

Variable	Baseline	Post-VLCD	Significance	Change (%)
Total body mass (kg)	103.9 ± 12.3	92.9 ± 9.6	*p* < 0.0001	− 10.4
BMI (kg/m^2^)	32.2 ± 4	28.9 ± 3.6	*p* < 0.0001	− 10.3
Lean body mass (kg)	65.2 ± 6	60.9 ± 4.8	*p* < 0.0001	− 6.4
Fat mass (kg)	35.3 ± 7.4	28.5 ± 6.9	*p* < 0.0001	− 19.7
Visceral fat (kg)	2.07 ± 0.8	1.5 ± 0.7	*p* < 0.0001	− 29.8

### Correlation between changes in fasting BCAA’s and HOMA-IR values

VLCD reduced HOMA-IR (1.2 ± 0.62 AU, *p* = 0.01; Fig. 3A). There was no correlation between changes in HOMA-IR and BCAA at baseline (Leu, R^2^: 2.498e−005, *p* = 0.98, Ile R^2^: 1.211−e006, *p* = 0.99, Val R^2^: 0.004, *p* = 0.85, sum BCAA R^2^ 0.001, *p* = 0.93), nor following VLCD (Leu, R^2^: 0.003, *p* = 0.86, Ile R^2^: 0.006, *p* = 0.82, Val R^2^: 0.02, *p* = 0.65, sum BCAA R^2^: 0.004, *p* = 0.85), Fig. 3B–E. To increase statistical power, the association between HOMA-IR and BCAA was investigated in an additional 32 individuals (total including OW participants, *N* = 58) to determine if a relationship existed, however Pearson’s correlation revealed no association (R^2^: 0.01, *p* = 0.31, Fig. 3F).

### Circulating BCAA in OW compared with NW volunteers

**Figure 1:**
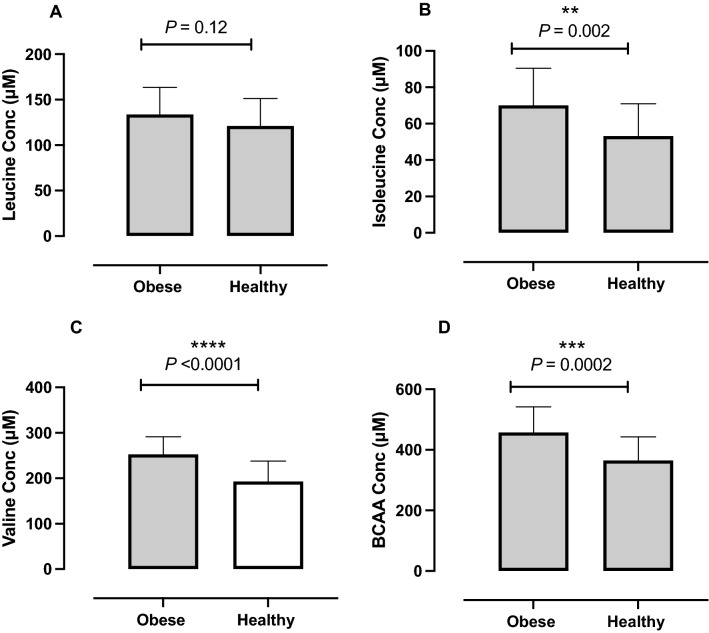
Comparison of individual BCAA’s and sum BCAA of OW (*n* = 26; mean BMI 32.2 ± 2.9 kg/m^2^) volunteers to NW, age-matched controls (*n* = 26; mean BMI 25.4 ± 3 kg/m^2^). There was a significant increase in total BCAA (**D**) in OW individuals compared to NW. The differences were driven primarily by isoleucine (**B**) and valine (**C**). Leucine (**A**) however remained unchanged.

**Figure 2:**
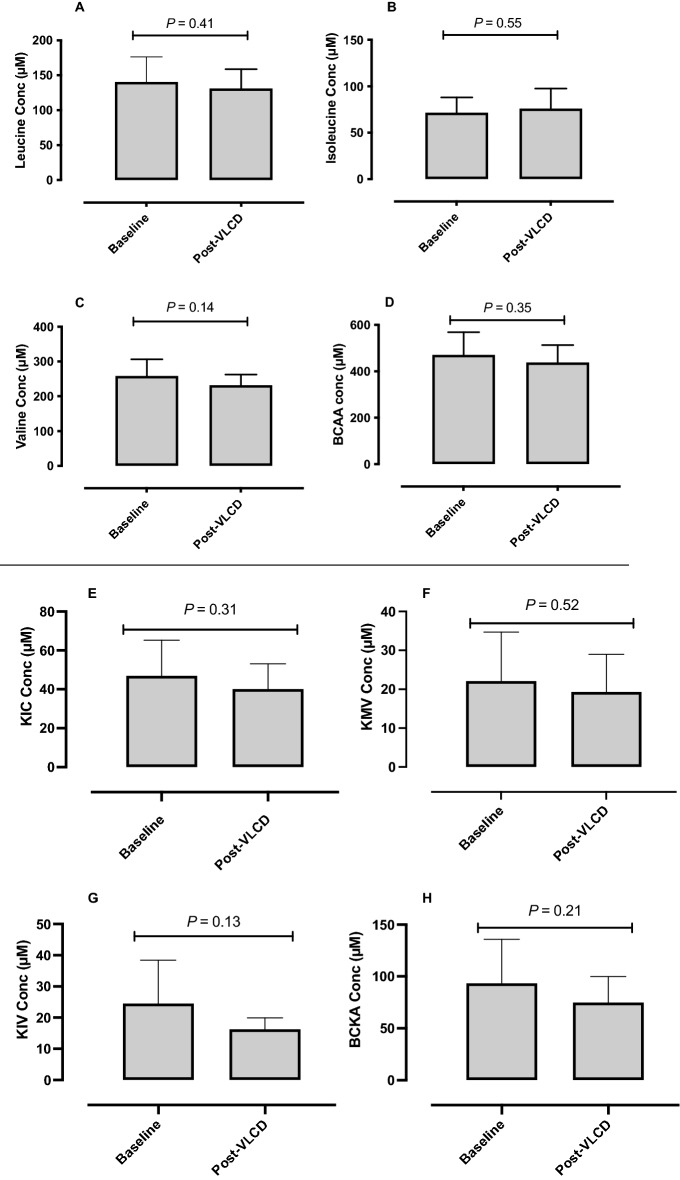
Baseline and post-VLCD comparisons of fasting plasma BCAA, and BCKA (*n* = 10). There were no differences in individual (**A**–**C**) or sum (**D**) BCAAs following 6-weeks VLCD. Similarly, there were no differences in individual (**E**–**G**) or sum (**H**) BCKA concentrations following VLCD.

**Figure 3:**
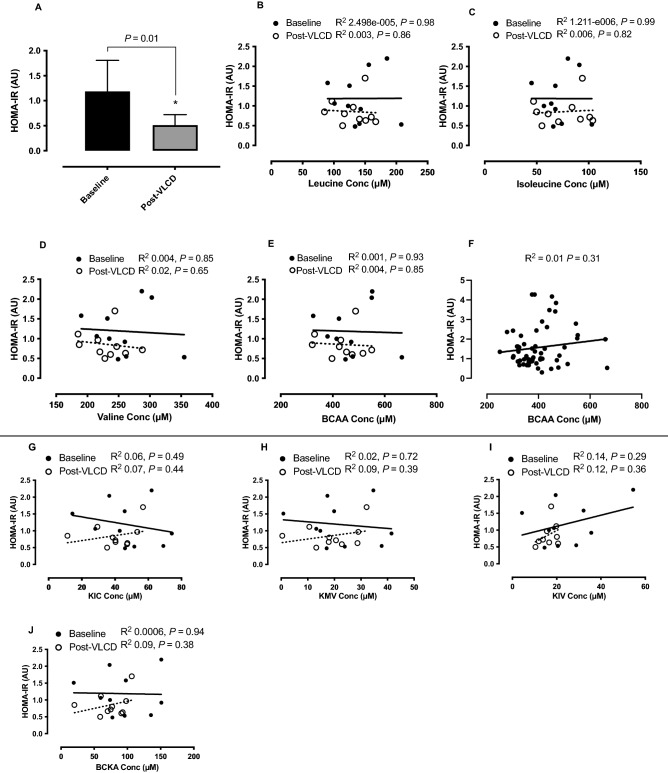
HOMA-IR (**A**) of individuals with obesity pre and post VLCD (A, *P* = 0.01) and correlations of HOMA-IR to individual and sum BCAA at baseline and post-VLCD (**B**–**E**). The baseline association between HOMA-IR and BCAA across a broader age and BMI range and mixed genders (**F**) with a greater *N* = 58 are displayed. The association between HOMA IR to individual BCKA (**G**–**I**) and total BCKA (**J**) are also displayed.

First, an unpaired *t-*test revealed no gender differences in baseline BCAA concentrations between our NW female (mean 349 ± 35 µM) and male (mean 372 ± 91 µM, *p* = 0.51) participants. BCAAs in our OW participants (457 ± 85 µM) were higher than in NW counterparts (365 ± 78 µM; *p* = 0.0002; Fig. 1D). Despite no changes in leucine concentrations (Fig. 1A), these differences were driven primarily by valine (OW 253 ± 38 µM, NW 193 ± 45 µM; *p* < 0.0001; Fig. 1C) and isoleucine (OW 70 ± 20 µM, NW 53 ± 18 µM; *p* = 0.002; Fig. 1B). Despite reductions in body weight, fat, and lean mass, as well as HOMA-IR, plasma BCAA concentrations were largely unchanged (baseline: total BCAA 471 ± 98 µM, leucine 141 ± 36 µM; isoleucine 71 ± 17 µM or valine 259 µM ± 47, Fig. 2A–D) following VLCD (sum BCAA 439 ± 75 µM, *p* = 0.35; leucine 131 ± 27 µM, *p* = 0.41; isoleucine 76 ± 22 µM, *p* = 0.55; or valine 231 ± 32 µM, *p* = 0.14). Similarly, BCKA concentrations at baseline (KIC 47 ± 18 µM, KMV 22 ± 13 µM, KIV 24 ± 14 µM, sum BCKA 94 ± 42 µM) were not significantly changed following six weeks of VLCD (KIC 40 ± 13 µM *p* = 0.31, KMV 19 ± 10 µM, *p* = 0.52, KIV 15 ± 4 µM, *p* = 0.13, sum BCKA 75 ± 25 µM, *p* = 0.21, Fig. 2E–H), nor were they associated with IS at baseline or following VLCD (Fig. 3G–J).

## Discussion

We investigated relationships between plasma BCAA/BCKA concentrations and measures of IR in OW men following a 6-week low calorie dietary intervention in the form of a ~ 600 kcal/day VLCD. Calorie restriction remains the most effective primary care step in treating obesity due to the rapid reduction in whole body mass and subsequent improvement in glycaemic control in both men and women^24,25^ The magnitude of weight loss (~ 11%) was comparable to that observed in other calorie restriction studies (~ 9.5–16%);^26,27^ and was also accompanied by significant reductions in HOMA-IR, fat mass, visceral fat and lean body mass—indicative of compliance with the intervention, assuring efficacy of VLCD.

Previously, robust correlations between IR and BCAA have been well-documented^4,10,11,28^, and BCAAs have been proposed as prognostic biomarkers for populations at risk of developing T2DM^5^. In accordance with numerous other studies^10,11,28,29^, including in monozygotic twins^30^, and including both men and women participants^11,31^, BCAAs were higher in our OW participants compared to those of a NW. Further, baseline comparison of our mixed gender NW cohort revealed no within group differences (*p* = 0.5), suggesting that plasma BCAA concentrations are unaffected by sex in healthy individuals and thus validating our test group comparison. Indeed, further modelling reveals that BCAA alone or in combination with other metabolites^5^, such as aromatic amino acids (AA;Phe/Tyr) and acylcarnitine species^11^ alanine and proline^32^ may hold value as metabolic ‘signatures’ of obesity and as predictors of T2DM. Nonetheless, relationships between plasma BCAAs and other prototype metabolic ‘signatures’ have been shown to vary across the lifespan. For example, elevated BCAAs are not associated with obesity in adolescents^33^, indicating that any relationship with plasma BCAAs may be context-dependant^29^. Furthermore, these associations have also been shown to differ between sexes. Reflecting this, in females, it was shown that BCAAs, phenylalanine, and the BCAA metabolite, 3-methyl-2-oxovalerate^34^ when combined revealed the strongest associations with IR. Conversely, in males it was BCAA, alanine, proline, glutamine and aromatic AAs that associated most strongly^32^. Therefore, elevations in BCAAs alone do not indicate underlying metabolic dysfunction and show variability across the lifespan and between genders. 

Contrary to previous observations between food over-consumption and higher BCAA^25^, we did not observe lowering of BCAA concentrations or in BCKAs, following 6-weeks of VLCD-induced weight loss. Our observations are in-line with some other VLCD studies of similar duration^35,36^ but contrast to other reports of BCAA lowering induced by calorie restriction induced weight loss following bariatric surgery^31,37^. While it is possible that individual subject characteristics may account for these contrasting results, other evidence suggests that reductions in BCAAs occur directly as a result of bariatric surgery per se*,* independent of weight loss. This may be due to increased BCAA catabolic gene expression in adipose tissue^38^, or due to the altered AA metabolism and reduced gastric capacity following operative procedures^39,40^. Therefore, while the causes of higher plasma BCAAs are not fully elucidated (i.e. the impact of age, caloric and protein intake, or impaired catabolism;^41,42^), our study suggests improvements in body composition and HOMA-IR occur independently of changes in fasting plasma BCAAs. Further, a relationship between BCAAs and IR was not apparent at baseline, in our OW participants, nor when we combined OW/NW cohorts (*n* = 58) to increase statistical power. This illustrates the lack of simplicity of relationships between plasma BCAAs and metabolic health; indeed in a previous study^43^, we demonstrated that fasting BCAAs positively correlate with lean mass and strength outcomes, as opposed to IR, following resistance exercise training.

Recent studies suggest a ‘clogging’ model of impaired BCAA catabolism, including increased accumulation of 3-hydroxyisobutyrate (3-HIB) a valine catabolite^42^. These authors reported increased muscle 3-HIB secretion in IR facilitates fatty acid uptake resulting in lipid accumulation^42^, a ‘synergistic’ relationship supported by other studies^13^. Genomic markers (e.g. reduced BCAA dehydrogenase complex and its regulatory enzyme protein phosphatase 1 K [PPM1K] in obese individuals)^44,45^ have also been proposed as possible mechanisms. In terms of BCAA catabolites, we measured each BCAA-derived BCKA as markers of altered BCAA metabolism. Similar to BCAAs, we observed neither a change in plasma BCKA concentrations (plasma biomarkers of muscle BCAA flux) or correlations between BCKAs and IS before/after VLCD. While of a preliminary nature, these findings do not support gross BCKA mis-handling in obese men. That being said, these data match those of BCAAs, thus suggesting more distal metabolites^42^ of BCAA metabolism may instead be implicated in tissue-level IR. Future work could adopt BCAA metabolomic and fluxomic approaches, including human muscle tissue, to address this. The lack of correlation between reductions in IR and BCAAs seen herein, despite improved metabolic health i.e. significant weight/ fat loss, suggests that reductions in BCAAs per se is not required to increase IS, but rather a metabolic reprogramming of insulin-sensitive tissue(s) in response to VLCD causes improvement in IS, preceding detectable changes in plasma BCAAs, particularly since no correlation was observed between total mass/fat loss and indices of IS.

The sample size of the VLCD cohort was a potential limitation of our study, future studies may benefit from enrolling a larger cohort in identifying robust links between plasma BCAAs and IR, as per the literature. An additional limitation of the present study may be the ‘low level’ IR of participants as a potential a confounding factor. HOMA-IR measures of ~ 3.5–5.5 have been reported in obesity^11,32^ compared to ~ 1.19 here. Furthermore, while VLCD elicited reductions in BMI (29 ± 4 kg/m^2^; ~ 10%), this improvement did not bring volunteers within the normal weight range (~ 25 kg/m^2^), with longer time-frames of VLCD likely required to achieve this^46^. A means to mitigate the loss of lean tissue or muscle mass in interventions of this nature are important to maximise the potential therapeutic benefits, with the increased protein intake being one such option. Undoubtedly, protective factors against IR and development of obesity/ T2DM include exercise participation since direct beneficial effects on β-cell function^47^ and liver function induced by exercise have been reported^48^, independent of changes in body mass.

In summary, despite VLCD resulting in weight loss and improved IS and despite higher BCAAs in OW participants, neither reductions in BCAAs nor correlations in BCAA/BCKA to HOMA-IR were observed following VLCD. We conclude that higher fasting BCAAs in obesity are unlikely a consequence of calorie over-consumption and are more likely to be genetically and/or metabolically programmed. Our findings do not rule out interventions aimed at driving BCAA/BCKA catabolism to improve IS in obesity/T2DM.

## Data Availability

The datasets used during the current study are available from the corresponding author on request.
